# Prevalence and associated factors of enamel developmental defects among Nigerian children with perinatal HIV exposure

**DOI:** 10.22514/jocpd.2023.007

**Published:** 2023-03-03

**Authors:** Nonso Emmanuel Onyia, Paul Akhigbe, Esosa Osagie, Ozoemene Obuekwe, Augustine Omoigberale, Vincent P. Richards, Modupe O. Coker, DOMHaIN Study team

**Affiliations:** 1 Department of Oral Pathology and Medicine, University of Benin, 302001 Edo State, Nigeria; 2 Research Department, Institute of Human Virology, Nigeria, 900107 Abuja, Nigeria; 3 Department of Oral and Maxillofacial Surgery, University of Benin, 300213 Benin City, Nigeria; 4 Child Health Department, University of Benin, 300213 Benin City, Nigeria; 5 Department of Biological Sciences, Clemson University, Clemson, SC 29631, USA; 6 Department of Oral Biology, Rutgers University, Newark, NJ 07103, USA

**Keywords:** HIV, Enamel, Opacity, Pediatric, Defects

## Abstract

To evaluate the prevalence and pattern of developmental defects of the enamel (DDE) and their risk factors among children born infected with Human Immunodeficiency Virus (HIV) and those born to HIV-infected mothers compared with their unexposed counterparts (*i.e.*, children born to uninfected mothers). This was an analytic cross-sectional study evaluating the presence and pattern of distribution of DDE in three groups of school-aged children (age, 4–11 years) receiving care and treatment at a Nigerian tertiary hospital, comprising: (1) HIV-infected (HI) on antiretroviral therapy (ART) (n = 184), (2) HIV-exposed but uninfected (HEU) (n = 186) and (3) HIV-unexposed and uninfected (HUU) (n = 184). Data capture forms and questionnaires were used to record the children’s medical and dental history based on clinical chart review and recall from their parents/guardians. Dental examinations were performed by calibrated dentists blinded to the study grouping. CD4+ (Cluster of Differentiation) T-cell counts were assayed for all participants. The diagnosis of DDE corresponded with the codes enumerated in the World Dental Federation’s modified DDE Index. Analyses relied on comparative statistics to determine risk factors associated with DDE. A total of 103 participants distributed among the three groups presented with at least one form of DDE, which indicated a prevalence of 18.59%. The HI group had the highest frequency of DDE-affected teeth (4.36%), while that of the HEU and HUU groups were 2.73% and 2.05%, respectively. Overall, the most encountered DDE was code 1 (Demarcated Opacity), accounting for 30.93% of all codes. DDE codes 1, 4 and 6 showed significant associations with the HI and HEU groups in both dentitions (*p* < 0.05). We found no significant association DDE and either very low birth weight or preterm births. A marginal association with CD4+ lymphocyte count was observed in HI participants. DDE is prevalent in school-aged children, and HIV infection is a significant risk factor for hypoplasia, a common form of DDE. Our results were consistent with other research linking controlled HIV (with ART) to oral diseases and reinforce advocacies for public policies targeted at infants exposed/infected perinatally with HIV.

## 1. Introduction

Developmental defects of the enamel (DDE) lead to irreversible changes in the structure and appearance of the teeth due to complex interactions between genetic and environmental factors [1]. The disturbances that cause these changes take place between 16 weeks of gestation and age of 5 [2] and occur as a result of disturbances in the apposition and mineralization of enamel matrix [3], leading to aesthetic disability, increased risk of dental caries and may ultimately lead to teeth loss.

Generally, the etiological factors attributed to DDE are categorized as hereditary or acquired. The constellation of the hereditary factors and components, including the patterns of inheritance, genetic anomalies and affected proteins, have been outlined [4]. The acquired or environmental etiological factors of DDE are infections; nutritional and neonatal disturbances such as jaundice [5]; maternal intoxication; hemolytic diseases; and cardiac, renal, and gastrointestinal illnesses [6]. Tolomeu *et al.* [7] reported links between gestational diabetes and DDE. Also, children born preterm and/or with very low birth weight (VLBW) have an increased risk for enamel hypoplasia compared with children born full term and/or with normal birth weight [8, 9].

In particular, HIV infection can lead to several systemic implications, including impairments in immune function in children, and can affect the development of dental enamel [10]. Similarly, the number of CD4 cells, an HIV categorizing marker, has been linked to the incidence and presentation of oral manifestations. With prevention of mother to child transmission (PMTCT) programs, treatment with antiretroviral therapy (ART) is often administered at an early age in children of HIV-infected mothers to control the infection. Despite being effective, ART might disrupt osteogenesis [11] and lead to DDEs in permanent dentition [10].

On a clinical basis, DDE is categorized into three fundamental types: demarcated opacity, diffused opacity and hypoplasia. Opacities, also termed hypomineralization, are alterations in translucency that manifest due to disturbances at the maturation phase, while hypoplasia, a quantitative defect, is initiated at the secretory phase of odontogenesis [12]. Conversely, molar incisor hypomineralization (MIH) is a site-specific presentation hypomineralization of one to four first permanent molars and can occasionally involve permanent incisors [13]. The modified DDE index proposed by World Dental Federation, Federation Dentaire Internationale (FDI) in 1992 is still largely employed for epidemiological studies, hence utilized in our present study to capture and simplify descriptions of other clinical entities such as MIH [14].

Several authors have previously reported links between developmental defects of the enamel and HIV infection and exposure [15, 16]. However, there are limited reports on the presentation of DDEs and their comparative relationships in HIV-infected children and their exposed-yet-uninfected counterparts. To address the knowledge gap in this field, we aimed to evaluate the prevalence and distribution pattern of enamel developmental defects among HIV-infected and exposed children compared with their unexposed counterparts. We hypothesized that perinatal HIV infection and exposure could increase the risk of DDE.

## 2. Materials and Methods

### 2.1 Study population and design

As part of the Centers for Disease Control and Prevention (CDC)-sponsored President’s Emergency Plan for AIDS Relief (PEPFAR) in Nigeria, tertiary sites are supported to care for people living with HIV (PLWH). Children aged 4–11 years were recruited as part of a cohort study from the pediatric PEPFAR outpatient clinic of the University of Benin Teaching Hospital (UBTH) who were HIV-infected (HI) and on antiretroviral therapy, children of HIV-infected parents who were exposed but not infected (HEU), and children who were unexposed and uninfected (HUU; controls). The results of this study were based on a cross-sectional analysis of DDE outcomes. This study was conducted over a period of one year.

### 2.2 Sample size estimation

This study was designed and powered to investigate the association between caries and HIV infection and exposure in children [17]. Based on an assumed prevalence of dental caries of 30%, a statistical significance and power of 0.05 and 0.80, respectively, a minimum sample of 276 participants (138 in each group) was required for a least detectable difference of 15% between HI and HUU children. Thus, a total of 568 children were included in the study. They comprised (1) HIV-infected (HI, n = 184), (2) HIV-exposed and uninfected (HEU, n = 186) and (3) HIV-unexposed and uninfected (HUU, n = 184) cases.

### 2.3 Selection criteria

All participants’ parents provided written informed consent to participate in the study after they completely understood the objectives of this study, which were duly explained to them. Children 8 years and above provided assent prior to study enrollment.

### 2.4 Examiners calibration

The calibration of the three examiners (dentists) was performed by a pediatric dentist. Prior to data collection, an extensive discussion session was held for differentiating between the various DDEs using clinical photographs showing enamel hypoplasia, molar incisor hypomineralization (MIH) and other hard tissue anomalies. A week after the session, the three dentists were each assessed independently using the same patients with different dental anomalies, which was done on three different occasions at weekly intervals. The results for each dentist were analyzed using Cohen’s Kappa statistics. The inter-examiner Kappa score was 0.91, and the intra-examiner values were 0.96, 0.92 and 0.84, respectively.

### 2.5 Study procedures

The study was conducted in the year 2019–2020 at UBTH, Benin City, Nigeria. Data collection was done in various phases: Medical records of the HI and HEU cases were obtained from perinatal records, which included birth weight and inception time of ART. Questionnaires were filled out by the parents/guardians. The questionnaire contained questions related to pregnancy, neonatal conditions, drug use, oral hygiene, dietary habits and the occurrence of primary diseases in childhood. CD4+ cell counts were obtained from laboratory assays.

#### 2.5.1 Dental examination

**FIGURE 1.**
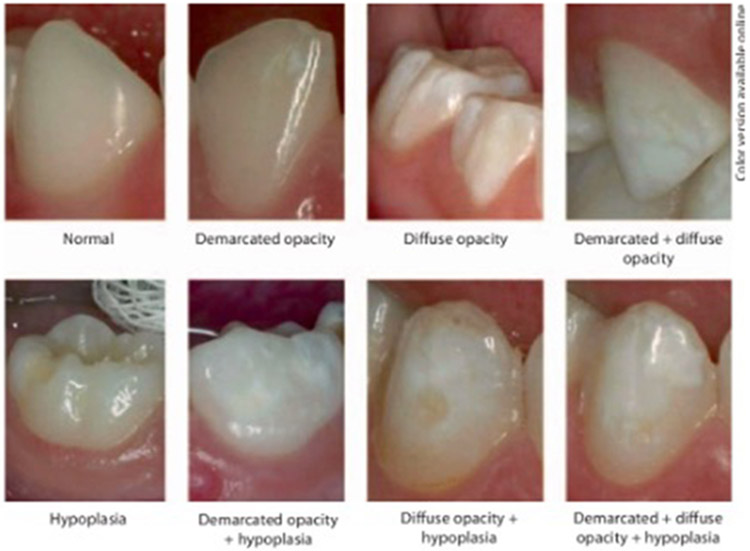
Clinical illustrations of the DDE codes developed from the Modified Developmental Defects of the Enamel Index (DDE Index) [18]

The patients were examined in a dental office under a direct lighting reflector using a dental mirror and probe. Enamel defects were diagnosed using the modified DDE Index recommended by the World Dental Federation (FDI Commission on Oral Health, Research and Epidemiology 1992) (Table 1 and Fig. 1). The index identifies and defines the type (hypoplasia, opacity, combination defects) and number (single, multiple) of tooth enamel defects on all primary and permanent teeth. The plaque was removed with gauze before DDE examination, and tooth surfaces were examined wet.

#### 2.5.2 Statistical analysis

The data collected were screened for completeness and analyzed using the IBM SPSS software (version 21.0, Armonk, NY, USA) Categorical variables are presented using frequencies and proportions. Bivariate analysis was performed using the chi-square test and Fisher’s exact test to determine associations between etiological factors and the presence of DDE. The level of statistical significance was set at *p* < 0.05, and the 95% confidence level was used to construct confidence intervals around point estimates.

## 3. Results

A total of 554 (HI, n = 184; HEU, n = 186; HUU, n = 184) children were examined (Table 2). There was no significant gender disparity between the study groups. Male participants accounted for 53.1% (n = 294) of all cases. A total of 103 participants distributed among the three groups (HI, n = 40; HEU, n = 33; HUU, n = 30) presented with at least one form of DDE, showing a prevalence of 18.59%. Thus, of the total 12,755 teeth examined, 388 (3.04%) teeth had DDE (Table 2).

Among the DDE-affected teeth, deciduous dentition accounted for 47.68% (n = 185). Among the investigated groups, HI had the highest frequency of DDE-affected teeth (4.36%), while that of the HEU and HUU groups were 2.73% and 2.05%, respectively (Table 2).

**FIGURE 2.**
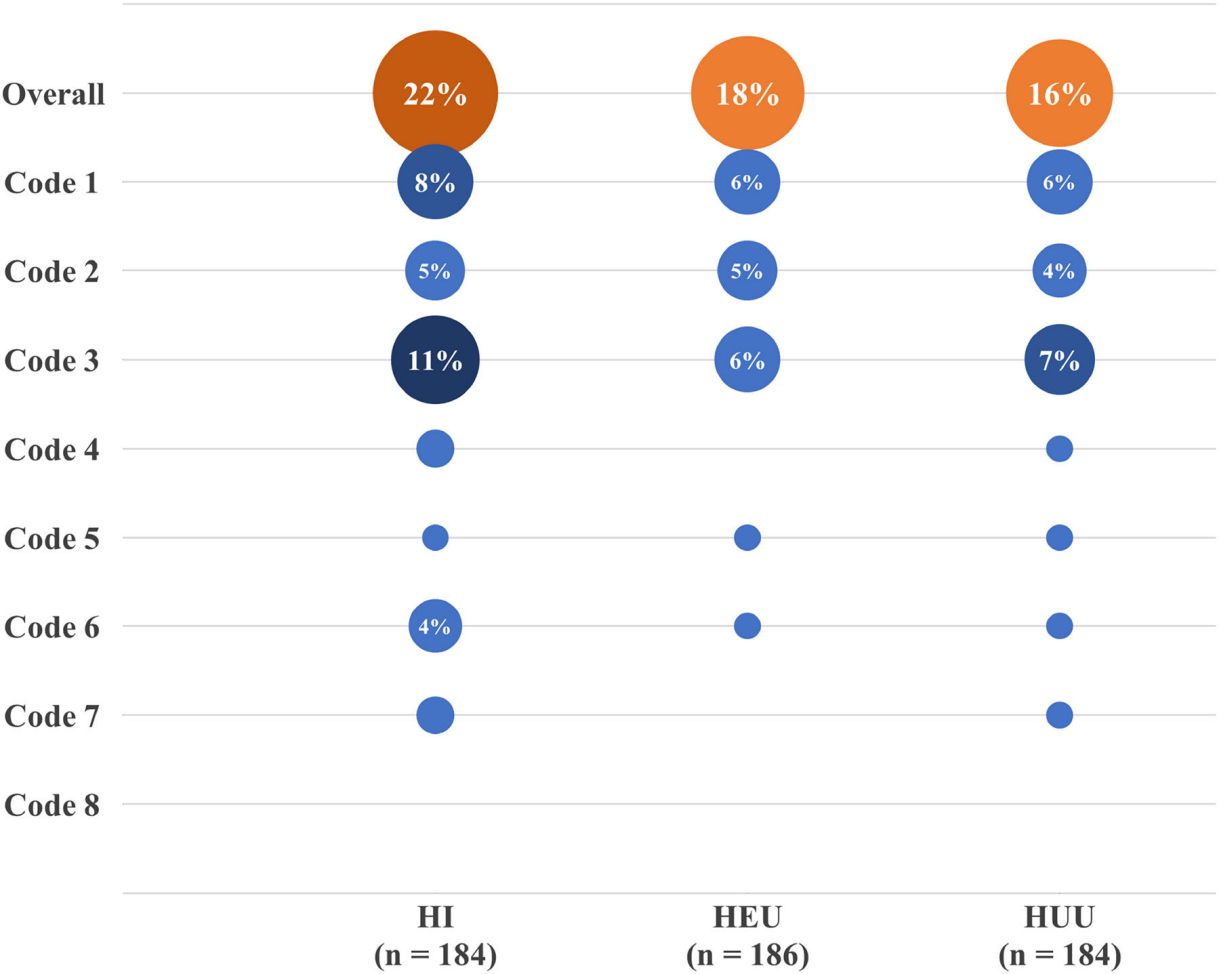
The combined prevalence of DDE codes by study groups. HI: HIV-infected; HEU: HIV-exposed but uninfected; HUU: HIV-unexposed and uninfected.

Overall, the most common DDE was code 1 (Demarcated Opacity), accounting for 30.93% of all codes. However, hypoplasia was observed cumulatively in codes 3, 6 and 7, representing 38.14% of the codes, and was distributed among 66 participants, representing 64.08% of children with at least one form of DDE (Fig. 2).

Comparing the presence of DDE among HI to the control group, HUU, we observed significant associations for DDE codes 1 and 6 compared to both dentitions (*p* < 0.05, Table 3). In examining the associations between HEU versus HUU, we observed a significant association for only DDE code 1 for both dentitions (*p* < 0.05, Table 3). In the deciduous dentition codes 1, 2, 3 and 4 were found to be more prevalent among the HI group. Conversely, in the permanent dentition, codes 1, 2 and 4 were more prevalent in the HEU group (Table 3).

Approximately one-fifth (n = 112) of all participants were born preterm (Table 4), and only 18.8% of them across all groups had one form of DDE, which showed a non-significant association with preterm birth (*p* > 0.05). There was no significant association between the presence of DDE with very low birth weight (VLBW). DDE was not significantly associated with CD4+ count at the time of examination (*p* = 0.69) in all participants. However, for HI children, there was a marginal association between low CD4 counts and DDE.

## 4. Discussion

To our knowledge, this study is the first to investigate HEU children in this context of elucidating the potential impact of perinatal HIV exposure on DDE. Our results suggest a significant relationship between DDE and HIV exposure and infection. Across the groups, the HI group had the highest frequency of DDE-affected teeth, while the least affected group was the HUU. Considering other associated perinatal factors, we observed weak evidence to suggest a significant association between DDE, preterm birth and VLBW in this study population.

Regional estimates of the prevalence of DDE in school-aged children are limited. Although the prevalence in this study, compared with a Nigerian epidemiological study involving 2015 children with a reported prevalence of DDE, was 11.2% [19], it varied widely with the prevalence of 72.5% and 33.2% in other related studies [20, 21]. The HI group had a higher prevalence of DDE than other groups, suggesting HIV as a risk factor for DDE [10, 15, 16]. Despite the relatively high frequency of DDE among the participants, we found that only 3.04% of the examined teeth had DDE. In the present study, we observed almost an equal distribution of DDE among deciduous (47.68%) and permanent teeth, which might be due to the age range of the participants who were predominantly in their mixed dentition stage. Prior reports on the prevalence of DDE in primary teeth are fewer than that of permanent dentition and reported a varying prevalence range from 10% to 49% [9, 22, 23]. Across the dentitions, molars were marginally more affected by DDE compared to incisors, with a ratio of 1.2:1. These finding coincides with two other reports [20, 24] and might be attributed to the fact that molars generally have later developmental period than incisors and could be affected additionally by perinatal circumstances such as preterm birth.

Previous studies reported opacities as the overall commonest type of DDE [20]. Lunardelli *et al.* [24] and Orenuga *et al.* [19] reported diffuse opacity as the prevalent DDE. On the contrary, we observed opacity (code 1) as the overall most prevalent DDE, which also coincides with the findings of Seow *et al.* [25] in an Australian population. In this study, the dominance of demarcated opacities was attributed to the permanent dentition when considered alone; however, hypoplasia (code 3 ) was the most prevalent among deciduous teeth, which was not observed in the study of Seow *et al.* [25], who reported a prevalence of opacities as three times that of hypoplasia in deciduous dentition.

We and others have reported an increased prevalence of dental caries in PLWH, and the data suggest an increased risk of caries due to HIV infection or ART [26-31]. Whether caries is a resulting comorbidity with living longer in PLWH on ART, is yet to be established. Salivary gland dysfunction [33], sugary formulations of pediatric ART syrups and suspensions [33], dysbiotic microbiota [28, 34, 35] and anomalies with tooth development [26] have been suggested as factors driving this increased risk in children. However, elucidating the strongest aetiologic factors driving caries susceptibility can guide and inform prevention programs, particularly as tooth development begins in-utero. Children perinatally exposed to HIV and ART are likely to experience deleterious peripartum effects on tooth structure, and therefore need to be targeted for caries prevention strategies.

In addition to examining HI children, we also examined HEU children. This is the first study to include an HEU cohort to investigate the distribution of DDE in comparison to HUU children. Generally, HEU children differ from their HUU counterparts in various ways. First, previous studies validated that HEU children have poorer health outcomes than HUU children [36-38]. Second, HEU children are more likely to have an overall perturbed growth. Jumare *et al.* [39] reported that HEU children had greater odds of being stunted and underweight than HUU in Jos, Nigeria. However, in this cross-sectional study, we could not consider the longitudinal effects of ART on the prevalence of DDE given the cross-sectional study design. Comparatively, earlier research showed that the timing of in-utero exposure to ART affected the growth measurements among HEU infants, with exposure from conception showing a reduced effect on infants’ length compared to exposure late in pregnancy [40].

Although speculative, it is reasonable to suggest that the HEU group could be more likely to have had disturbances at different stages of tooth development, possibly due to maternal illnesses, dietary inadequacies, drug irritants and other perinatal events. DDEs with a similar description might not be necessarily caused by similar etiological factors. Of note, the same etiological factor can produce different defects at different stages of tooth development [41]. Additionally, DDE may result from long-term biologic and environmental factors linked to etiologic and pathogenic processes [41]. This may explain the differential distributions of the DDE subtypes among the three study groups in the current study.

There was a weak association between preterm birth and VLBW with DDE, irrespective of the code in this study, which does not align with previous studies that reported a link between VLBW and increased prevalence of DDE [6, 42, 43]. This could be explained by the uniqueness of our present study population due to the inclusion of children born to HIV-infected and uninfected mothers who received care and treatment in a tertiary healthcare facility and could also explain the low prevalence of preterm birth and VLBW.

The association of CD4+ T-lymphocytes counts with DDE has been scarcely reported. A review by Atar *et al.* [15] established links between low CD4+ count and increased likelihood of oral manifestations in HIV patients [15], however, Pontes *et al.* [10] specifically reported a non-significant association between low CD4 count and DDE, similar to the findings of our present study.

Further, our results highlight that the long-lasting impacts of developmental defects of the enamel in primary and permanent dentitions must not be ignored. To our knowledge, this is the largest study in Nigeria that describes the association of DDE with perinatal HIV exposure and HIV-1 infection among children. As previously mentioned, the strength of the present study is the inclusion of the HEU group in comparative discussions of oral presentations attributable to HIV exposure without infection. Further, it clarifies the potential impact of perinatal HIV exposure in-utero in the context of ART prophylaxis to prevent mother-to-child transmission. Additional studies are needed to clarify the interplay between maternal HIV and dental, oral and craniofacial anomalies (DOC) to elucidate modifiable risk factors for their treatment and prevention.

However, there were some limitations. Considering that this was a cross-sectional study, we could not address the long-term impact of maternal HIV infection. However, we believe the temporary effect of HIV exposure can be assessed within this design. Also, the data collected could not isolate, the longitudinal effects of antiretroviral therapy on presentations of DDE among the HI and HEU groups. Although a previous study reported that the prevalence of DDE did not differ by tenofovir-based regimen [44], another study observed an association between the use of antiretroviral regimens with protease inhibitors or efavirenz and DDE in cases with permanent dentitions [10]. Further, the independent effect of sociodemographic factors, such as dietary history and maternal infections, were not addressed in this study due to incomplete data. Despite these limitations, the present study forms a locus for further quantitative and longitudinal studies on oral manifestations attributable to HIV exposure, could stimulate clinical vigilance amongst pediatricians and reinforces advocacies for public health policies targeted at infants exposed/infected perinatally with HIV. We strongly recommend future related studies to test the epigenetic consequences of HIV exposure on developmental anomalies of the teeth and the orofacial region.

## 5. Conclusions

HIV infection and exposure are significant risk factors for DDE among school-aged children. This present study showed that demarcated opacity and hypoplasia were the commonest forms of DDE amongst children with HIV infection and perinatal exposure.

## Figures and Tables

**Table 1.** Classification of Developmental Enamel Defects (DDEs), According to the Modified Developmental Defects of Enamel Index (DDE Index).

Code	Condition^a^/Combination^b^
0	Normal
1	Demarcated opacity
2	Diffuse opacity
3	Hypoplasia
4	Other defects
5	Demarcated and diffuse opacity
6	Demarcated opacity and hypoplasia
7	Diffuse opacity and hypoplasia
8	All three deficiencies (demarcated, diffuse opacity and hypoplasia)

a Codes 0–3 correspond to the conditions listed.

b Codes 5–8 correspond to the combination.

**Table 2.** Sociodemographic characteristics of the participants and their DDE profile.

Characteristics		HI n(%) (n = 184)	HEU n(%) (n = 186)	HUU n(%) (n = 184)	Total
Age group					
	4–7 years	71 (39%)	91 (49%)	76 (41%)	238
	8–11 years	113 (61%)	95 (51%)	108 (59%)	316
Gender					
	Male	89 (48%)	100 (54%)	105 (57%)	294
	Female	95 (52%)	86 (46%)	79 (43%)	260
Participants with DDE					
	Yes	40 (21.74%)	33 (17.74%)	30 (16.30%)	103
	No	144 (78.26%)	153 (82.26%)	154 (83.70%)	451
Total number of teeth examined		4218	4285	4252	12,755
Total number of teeth with DDE		184 (4.36%)	117 (2.73%)	87 (2.05%)	388

HI: HIV-infected; HEU: HIV-exposed but uninfected; HUU: HIV-unexposed and uninfected; DDE: developmental defects of the enamel.

**Table 3.** Distribution of DDE codes among various dentitions.

	Primary Teeth					Permanent Teeth				
	HI (n = 184)	HEU (n = 186)	HUU (n = 184)	*p* value HI *vs.* HUU	*p* value HEU *vs.* HUU	HI (n = 184)	HEU (n = 186)	HUU (n = 184)	*p* value HI *vs.* HUU	*p* value HEU *vs.* HUU
Present	27 (15%)	18 (10%)	17 (9%)	0.11	0.16	18 (10%)	21 (11%)	15 (8%)	0.58	0.31
Absent	157 (85%)	168 (90%)	167 (91%)			166 (90%)	165 (89%)	169 (92%)		
1. Demarcated Opacity	17 (9%)	2 (1%)	10 (5%)	<0.01	0.02	36 (20%)	41 (22%)	14 (8%)	<0.01	<0.01
2. Diffuse Opacity	17 (9%)	6 (3%)	11 (6%)	0.01	0.30	11 (6%)	26 (14%)	20 (11%)	0.10	0.17
3. Hypoplasia	45 (24%)	22 (12%)	19 (10%)	<0.01	0.62	9 (5%)	5 (3%)	7 (4%)	0.61	0.54
4. Other defects	7 (4%)	0 (0%)	0 (0%)	0.01	-	5 (3%)	0 (0%)	1 (1%)	0.10	0.01
5. Demarcated & diffuse	1 (1%)	4 (2%)	1 (1%)	1.00	0.18	4 (2%)	6 (3%)	0 (0%)	0.04	0.01
6. Demarcated & hypoplasia	13 (7%)	5 (3%)	1 (1%)	<0.01	0.10	14 (8%)	0 (0%)	1 (1%)	<0.01	0.31
7. Diffuse & hypoplasia	3 (2%)	0 (0%)	0 (0%)	0.08	-	2 (1%)	0 (0%)	2 (1%)	1.00	0.15
8. All three defects	0 (0%)	0 (0%)	0 (0%)	-	-	0 (0%)	0 (0%)	0 (0%)	-	-

HI: HIV-infected; HEU: HIV-exposed but uninfected; HUU: HIV-unexposed and uninfected.

**Table 4.** Association between risk factors and DDE.

Factors		HI			HEU			HUU		
		DDE Present	DDE Absent	*p* Value	DDE Present	DDE Absent	*p* Value	DDE Present	DDE Absent	*p* Value
Preterm birth (<37 weeks gestational age)										
	Yes	8	25	0.70	7	33	0.96	6	33	0.86
	No	32	119		26	120		24	121	
VLBW (<1500g)										
	Yes	4	14	0.96	0	2	0.51	0	1	0.67
	No	36	130		33	151		30	153	
Low CD4 count ≤500 cells/μL										
	Yes	9	53	0.09	33	153	-	30	154	
	No	31	91		0	0		0	0	

HI: HIV-infected; HEU: HIV-exposed but uninfected; HUU: HIV-unexposed and uninfected; DDE: developmental defects of the enamel; VLBW: very low birth weight; CD4: Cluster of differentiation 4 (CD4) lymphocyte counts.

## ABBREVIATIONS

ART: Antiretroviral therapy
CD4 cells: Clusters of differentiation 4 glycoproteins associated with T cell receptors, especially on the surface of helper T cells
DDE: Developmental defects of the enamel
DOMHaIN: Dental caries and its association with oral microbiomes and HIV in young children—Nigeria
HIV: Human immunodeficiency virus
PEPFAR: President’s Emergency Plan for AIDS Relief
PLWH: People living with HIV
VLBW: Very low birth weight
